# The effect of hyperbaric oxygen therapy on myocardial function in post-COVID-19 syndrome patients: a randomized controlled trial

**DOI:** 10.1038/s41598-023-36570-x

**Published:** 2023-06-10

**Authors:** Marina Leitman, Shmuel Fuchs, Vladimir Tyomkin, Amir Hadanny, Shani Zilberman-Itskovich, Shai Efrati

**Affiliations:** 1 Department of Cardiology, Shamir Medical Center, Zerifin, Israel; 2 Sagol Center for Hyperbaric Medicine and Research, Shamir Medical Center, Zerifin, Israel; 3 Sackler School of Medicine, Tel Aviv University, Tel Aviv, Israel; 4 Sagol School of Neuroscience, Tel Aviv University, Tel Aviv, Israel

**Keywords:** Cardiology, Echocardiography, Population screening

## Abstract

Post-COVID-19 condition refers to a range of persisting physical, neurocognitive, and neuropsychological symptoms following SARS-CoV-2 infection. Recent evidence revealed that post-COVID-19 syndrome patients may suffer from cardiac dysfunction and are at increased risk for a broad range of cardiovascular disorders. This randomized, sham-control, double-blind trial evaluated the effect of hyperbaric oxygen therapy (HBOT) on the cardiac function of post-COVID-19 patients with ongoing symptoms for at least three months after confirmed infection. Sixty patients were randomized to receive 40 daily HBOT or sham sessions. They underwent echocardiography at baseline and 1–3 weeks after the last protocol session. Twenty-nine (48.3%) patients had reduced global longitudinal strain (GLS) at baseline. Of them, 13 (43.3%) and 16 (53.3%) were allocated to the sham and HBOT groups, respectively. Compared to the sham group, GLS significantly increased following HBOT (− 17.8 ± 1.1 to − 20.2 ± 1.0, *p* = 0.0001), with a significant group-by-time interaction (*p* = 0.041). In conclusion, post-COVID-19 syndrome patients despite normal EF often have subclinical left ventricular dysfunction that is characterized by mildly reduced GLS. HBOT promotes left ventricular systolic function recovery in patients suffering from post COVID-19 condition. Further studies are needed to optimize patient selection and evaluate long-term outcomes.

This study was registered with ClinicalTrials.gov, number NCT04647656 on 01/12/2020.

## Introduction

The severe acute respiratory syndrome coronavirus 2 (SARS-CoV-2) pandemic has resulted in more than 541 million infected cases, as of June 2022. Even though most infected patients recover, around 10–30% remain with persistent symptoms that can significantly affect their quality of life^1,2^. The post-COVID-19 syndrome is defined by the World Health Organization as having physical, neurocognitive, and psychiatric symptoms three months after a confirmed onset of COVID-19 that persists for more than two months and cannot be explained by an alternative diagnosis^1^.

The long-term impact of COVID-19 associated cardiac injury has recently been revealed. A radiological study of 100 discharged COVID-19 patients found cardiac abnormalities and myocardial inflammation in 78% and 60% of participants, respectively, which were not associated with the initial COVID-19 severity^3^. Rajpal et al. found signs of myocardial inflammation using magnetic resonance imaging (MRI) performed on 26 college athletes with asymptomatic SARS-CoV-2 infection^4^. Abnormalities of left ventricular remodeling were found in 29% of 79 COVID-19 survivors in echocardiography examinations performed three months post-discharge^5^. Al-Aly et al. using a large cohort of 153,760 patients, demonstrated that COVID-19 survivors are at an increased risk of a broad range of cardiovascular disorders including cerebrovascular disorders, dysrhythmias, ischemic and non–ischemic heart disease, pericarditis, myocarditis, heart failure, and thromboembolic disease^6^.

Currently, treatment options for post-COVID-19 condition include targeted anti-inflammatory molecules and specific diets. However, none have been determined effective^7–9^. Moreover, no specific therapy has been suggested for cardiovascular manifestations.

In recent years, evidence has accumulated about hyperbaric oxygen therapy’s (HBOT) efficacy^10–18^. HBOT includes the inhalation of 100% oxygen at pressures exceeding 1 atmosphere absolute, thus enhancing the amount of oxygen dissolved in the body tissues. This combined action of hyperoxia and hyperbaric pressure leads to significant improvement in tissue oxygenation while targeting both oxygen and pressure sensitive genes^10^. Recently, Robbins et al. suggested a possible benefit on both fatigue and cognitive function with HBOT in a recent case series of ten post-COVID-19 condition patients^19^. To evaluate the effect of HBOT on post-COVID-19 syndrome, we designed a double-blinded randomized controlled trial. We found significant improvement in both cognitive function, and physical and psychiatric symptoms^20^. Complementing that clinical trial, cardiac function was evaluated.

The aim of the current study was to evaluate the effects of HBOT on cardiac function in patients suffering from the post COVID-19 syndrome in a randomized, sham-control, double-blind clinical trial.

## Methods

### Patients

Included patients were over 18 years old with a reported post-COVID-19 cognitive symptoms that affected their quality of life and persisted for more than three months after an RT-PCR confirmed mild-moderate symptomatic SARS-CoV-2 infection. Patients were excluded if they had a history of pathological cognitive decline, traumatic brain injury, or any other known non-COVID-19 brain pathology.

### Design

The current study is part of phase II exploratory clinical trial. A prospective randomized, double-blind, sham-controlled study was conducted from December 14, 2020, to December 27, 2021, at Shamir Medical Center (SMC), Israel. After signing informed consent, patients were randomized to either HBOT or sham-control groups in a 1:1 ratio according to a computerized randomization table, performed using an in-house software written in MATLAB R2021b (MathWorks, Natick, MA), supervised by a blinded researcher.

Patients were questioned after the first session on their perception regarding the treatment they received to evaluate masking. The evaluation procedure was done at baseline and 1–3 weeks after the last HBOT/sham session. All evaluators were blinded to the patients’ group allocations. The study was approved by SMC’s Institutional Review Board (IRB) (No. 332–20-ASF) and all participants signed informed consent before their inclusion. All research was performed according to the relevant guidelines and regulations. This study was registered with ClinicalTrials.gov, number NCT04647656 on 01/12/2020. The primary end point of this clinical trial was cognitive function which was reported in the first publication along with secondary endpoints of the associated related symptoms brain MRI features. This article focused on the cardiac functions evaluated on the same study population.

### Intervention

Both HBOT and sham protocols were administrated in a multi-place Starmed-2700 chamber (HAUX, Germany). The protocol comprised of 40 daily sessions, five sessions per week within a two-month period. The HBOT protocol included breathing 100% oxygen by mask at 2ATA for 90 min with five-minute air breaks every 20 min. Compression/decompression rates were 1.0 m/minute. The sham protocol included breathing 21% oxygen by mask at 1.03 ATA for 90 min. To mask the controls, the chamber pressure was raised to 1.2 ATA during the first five minutes of the session along with circulating air noise followed by decompression (0.4 m/minute) to 1.03 ATA during the next five minutes.

All the patients underwent an echocardiography examination twice, at baseline (before the first intervention session) and 1–3 weeks post the last protocol session.

### Primary and secondary outcomes

#### Primary outcomes—Global Longitudinal Strain

Secondary outcomes: myocardial work index parameters: Global Work Index, Global Constructive Work. Global Wasted Work, Global Work Efficacy.

### Echocardiography examination

All echocardiography exams were performed using Vivid E95, (General Electric; Horten, Norway) with a standard transducer of 1.7–4 Hz. The frame rate during echocardiography examinations was greater than or equal to 40 frames per second. Comprehensive transthoracic echocardiography examinations were performed according to the latest recommendations on chamber quantification^21^. Briefly, linear, volumetric, and Doppler measurements were performed. Standard echocardiographic views were acquired: parasternal long and short axis at three levels: basal, mid-ventricular and apical, apical 4-chamber, 2-chamber, and 3-chamber views. Diastolic function was assessed according to the current recommendations^22^: E wave amplitude, A wave amplitude, E/A ratio, E wave deceleration time, tissue Doppler E' septal velocity [E's], and tissue Doppler E' lateral velocity [E'l].

Biplane left atrial volume index (LAVi) was calculated according to the following formula:

$$\frac{8}{3}\uppi *\frac{\left(\mathrm{A}1\right)*\left(\mathrm{A}2\right)}{L}$$

where A1 and A2 are the area of the left atrium obtained from apical 4- and 2-chamber views respectively, and L is the shortest vertical size of the left atrium. LAVi was normalized for body surface area.

Left ventricular mass index (LVMi) was calculated according to the following formula:

$$0.8* 1.4*\left[ {\left( {{\text{IVS}} + {\text{PW}} + {\text{LVID}}} \right)^{3} - {\text{LVID}}^{3} } \right] + 0.6\,{\text{g}}$$

where IVS is the interventricular septal at end-diastole, PW is the posterior wall thickness at end-diastole and LVID is the left ventricle at end-diastole. LVMi was normalized for body surface area.

All echocardiography examinations were then transferred to the EchoPAC workstation (Version 204), for further off-line speckle-tracking imaging analysis. Speckle tracking imaging analysis was done according to the original recommendations from the apical views^23^. The aortic valve closure on the apical long axis view served as a reference for end-systole and was verified by aortic Doppler flow recorded from the apical five-chamber view^23^. Off-line speckle tracking analysis with calculations of global longitudinal strain (GLS) and myocardial work index (MWI) variable were performed^24^. Speckle-tracking imaging analysis was performed by a senior cardiologist experienced in echocardiography (ML). Twenty random patients were evaluated independently by both M.L. and V.T. for inter- and intra-observer variability.

### Statistical analysis

Continuous data were expressed as means ± standard deviations (SD). Normality assumption was evaluated according to a Kolmogorov–Smirnov test. A paired t-test and a two-sample Student’s t-test were used to compare means as appropriate. A Welch's *t*-test was used for unequal variances*.* To evaluate HBOT’s effect, a mixed-model repeated-measure ANOVA model was used to compare post-treatment and pre-treatment data. The model included time, group, and group-by-time interaction. The 95% confidence interval was used, and a *p* value < 0.05 was considered significant. Categorical data are expressed in numbers and percentages. Univariate analysis was performed using chi-square/Fisher's exact test (where appropriate). Data analysis was performed using IBM SPSS Statistics for Windows, Version 28.0. (Armonk, NY: IBM Corp). A sample size for this study was defined previously^20^.

## Results

**Figure 1:**
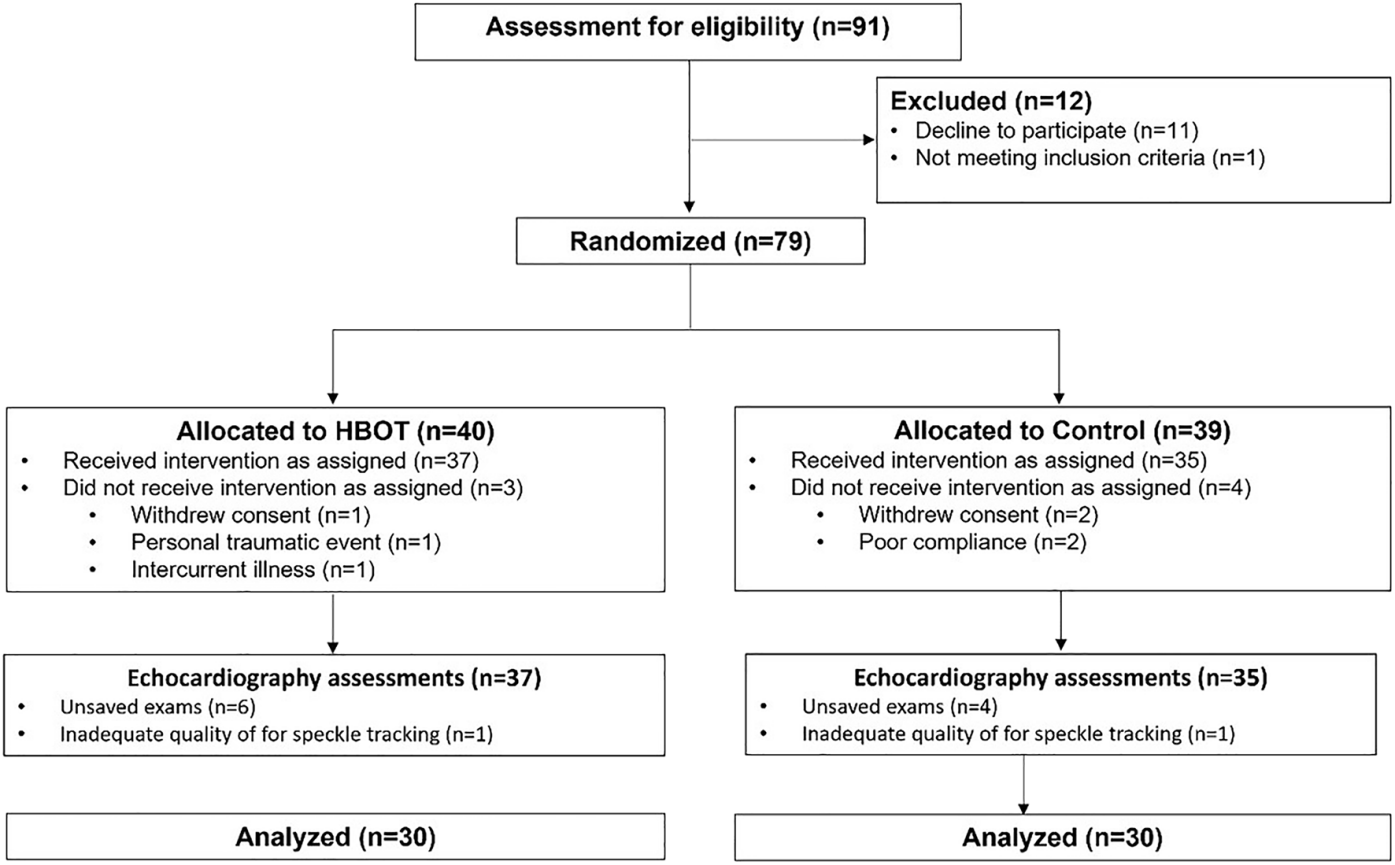
Patient flowchart.

Out of 91 patients eligible to participate in the study, 11 patients did not complete the baseline evaluation and one patient did not meet inclusion criteria. Thus, 79 patients were randomized to either HBOT or sham arms. Two patients from the sham group withdrew their consent during treatment, and two patients did not complete the post-protocol assessments due to poor compliance. From the HBOT group, three patients did not complete post-protocol assessments due to intercurrent illness, a personal traumatic event, and withdrawal of consent. Accordingly, 37 patients from the HBOT group and 35 patients from the sham group completed the protocol and underwent post-treatment assessments. Six echocardiography examinations from the HBOT group and 4 from the sham group were not transferred appropriately and saved for analysis. One exam from each group was not suitable for speckle tracking imaging analysis due to suboptimal quality. Figure 1 shows the echocardiography examinations of the 30 patients from HBOT and the 30 patients from the sham group that were included in the final analysis.

Patient baseline characteristics are detailed in Table 1. Mean patient age was 48.3 ± 10.5 and 45.6 ± 9.1 years old in the treatment and sham groups, respectively (*p* = 0.30). There were 15/15 (50%) and 10/20 (33.3%) males in the treatment and the sham groups, respectively, (*p* = 0.20). None of the patients had a history of known heart failure. No statistically significant differences between the two groups were observed in baseline characteristics.

**Table 1:** Patient characteristics.

	HBOT	Sham	*p* value
N	30	30	1.00
Age, years	48.3 ± 10.5	45.6 ± 9.1	0.30
Male/Female	15/15	10/20	0.20
BMI	27.1 ± 5.1	25.4 ± 4.3	0.17
BSA, m^2^	1.9 ± 0.25	1.8 ± 0.1.0	0.35
Time from in infection, days	153.9 ± 74.7	161.8 ± 59.6	0.65
Hospitalized	4 (13.3%)	4 (13.3%)	1.00
Hypertension	4 (13.3%)	2 (6.7%)	0.39
Chronic pulmonary disease	2 (6.7%)	1 (3.3%)	0.56
Previous smoking	8 (26.7%)	6 (20%)	0.55
Previous PCI	1 (3.3%)	0	0.16
ACE inhibitors	1 (3.3%)	1 (3.3%)	1.00
Beta-blockers	1 (3.3%)	1 (3.3%)	1.00
Calcium channel blockers	1 (3.3%)	1 (3.3%)	1.00
Thiazide diuretics	1 (3.3%)	1 (3.3%)	1.00
Antiplatelets	2 (6.7%)	0	0.49
Statins	1 (3.3%)	1 (3.3%)	1.00
Symbicort (Budesonide/formoterol 160/4.5 mcg/dose)	1 (3.3%)	1 (3.3%)	1.00
Anxiolytics	1 (3.3%)	2 (6.7%)	1.00
Proton pump inhibitors	3 (10%)	4 (13.3%)	1.00

BMI, body mass index; BSA, body surface area; PCI, percutaneous coronary interventions; ACE, angiotensin converting enzyme.

Conventional echocardiography parameters including ejection fraction (EF), LAVi, LVMi, diastolic function, and pulmonary artery pressure were similar between both groups (*p* > 0.05), (Table 2).

**Table 2:** Baseline echocardiography and hemodynamic parameters.

	HBOT	Sham	*p* value
N	30	30	
HR 1, beats/minute	68 ± 11	64 ± 10	0.08
HR 2, beats/minute	65 ± 12	66 ± 12	0.67
SBP 1, mmHg	126.7 ± 18	116.9 ± 20.2	0.1
SBP 2, mmHg	122 ± 16.5	117.0 ± 14.7	0.9
DBP 1, mmHg	73.6 ± 10.9	69.3 ± 11.7	0.15
DBP 2, mmHg	68.8 ± 9.6	68.0 ± 13.9	0.8
EF 1, %	59.2 ± 5.1	61.0 ± 5.0	0.18
EF 2, %	60.1 ± 4.7	60.8 ± 5.2	0.6
LAVi 1, ml/m^2^	27.3 ± 5.4	26.5 ± 6.0	0.61
LAVi 2 ml/m^2^	27.2 ± 6.4	24.2 ± 6.4	0.08
LVMi 1, kg/m^2^	75.4 ± 15.2	67.8 ± 16.9	0.16
LVMi 2, kg/m^2^	73.3 ± 13.6	72.0 ± 13.2	0.71
E/E’ 1	7.4 ± 2.4	6.8 ± 1.7	0.25
E/E’ 2	7.5 ± 2.3	6.7 ± 1.5	0.1
PAP 1, mmHg	26.7 ± 5.9	23.4 ± 5.8	0.38
PAP 2, mmHg	25.6 ± 4.8	25.4 ± 4.6	0.08

HR, heart rate; SBP, systolic blood pressure; DBP, diastolic blood pressure; LAVi, left atrial volume index; PAP, pulmonary artery pressure.

Speckle tracking imaging parameters are presented in Table 3. There were no significant differences between the two groups at baseline while GLS was mildly reduced at baseline (with a normal GLS − 20%) in both groups (− 19.1% and − 19.5%, *p* = 0.29). Post-HBOT, there was a statistically significant elevation of GLS (− 19.1 ± 1.8% to − 20.4 ± 2.1, *p* = 0.01) compared to the sham group (− 19.5 ± 2.1 to − 20.0 ± 2.1, *p* = 0.27). The net effect size was 0.268, and the mixed model analysis was not significant (*p* = 0.237) (Table 3). There were no other significant changes between the two groups (Table 3).

**Table 3:** Myocardial work index changes.

	HBOT (N = 30)			Sham (N = 30)			*p* value *	ANOVA group by time interaction		
	Baseline	post	*p* value	Baseline	post	*p* value		Net size effect	F	*p* value
GLS, %	− 19.1 ± 1.8	− 20.4 ± 2.1	**0.01**	− 19.5 ± 2.1	− 20.0 ± 2.1	0.27	0.29	0.268	1.463	0.237
GWI, mmHg'	1981.8 ± 390.9	2043.8 ± 346.1	0.54	1836.4 ± 342.0	1911.6 ± 416.6	0.50	0.19	0.302	0.001	0.975
GCW, mmHg'	2279.1 ± 423.8	2330.8 ± 348.8	0.63	2158.2 ± 377.1	2191.4 ± 475.5	0.78	0.30	0.275	0.040	0.842
GWW, mmHg'	60.1 ± 22.8	54.7 ± 21.3	0.39	54.4 ± 35.1	56.3 ± 42.6	0.78	0.48	0.359	0.513	0.481
GWE, %	96.8 ± 1.0	97.1 ± 0.89	0.2	96.9 ± 1.59	96.9 ± 1.73	0.24	0.78	0.262	0.419	0.523

GLS, global longitudinal strain; GWI, global work index; GCW, global constructive work; GWW, global wasted work; GWE, global work efficacy.

*p—the significance of baseline differences.

Significant values are in bold.

**Figure 2:**
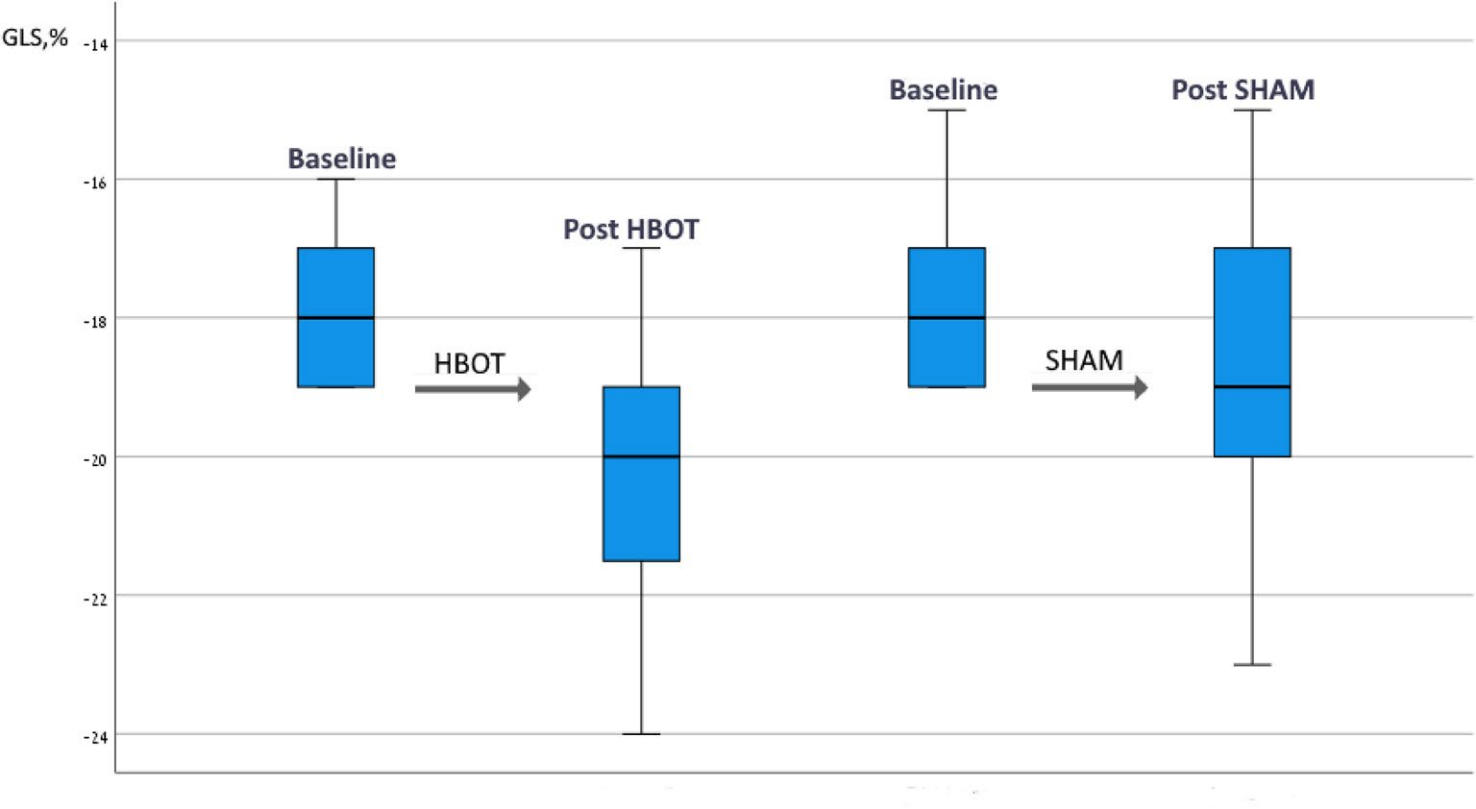
Global longitudinal strain in the group of patients with reduced strain at baseline. GLS improved in both groups of patients with reduced strain at baseline. In patients that underwent HBOT, GLS improved significantly.

**Figure 3:**
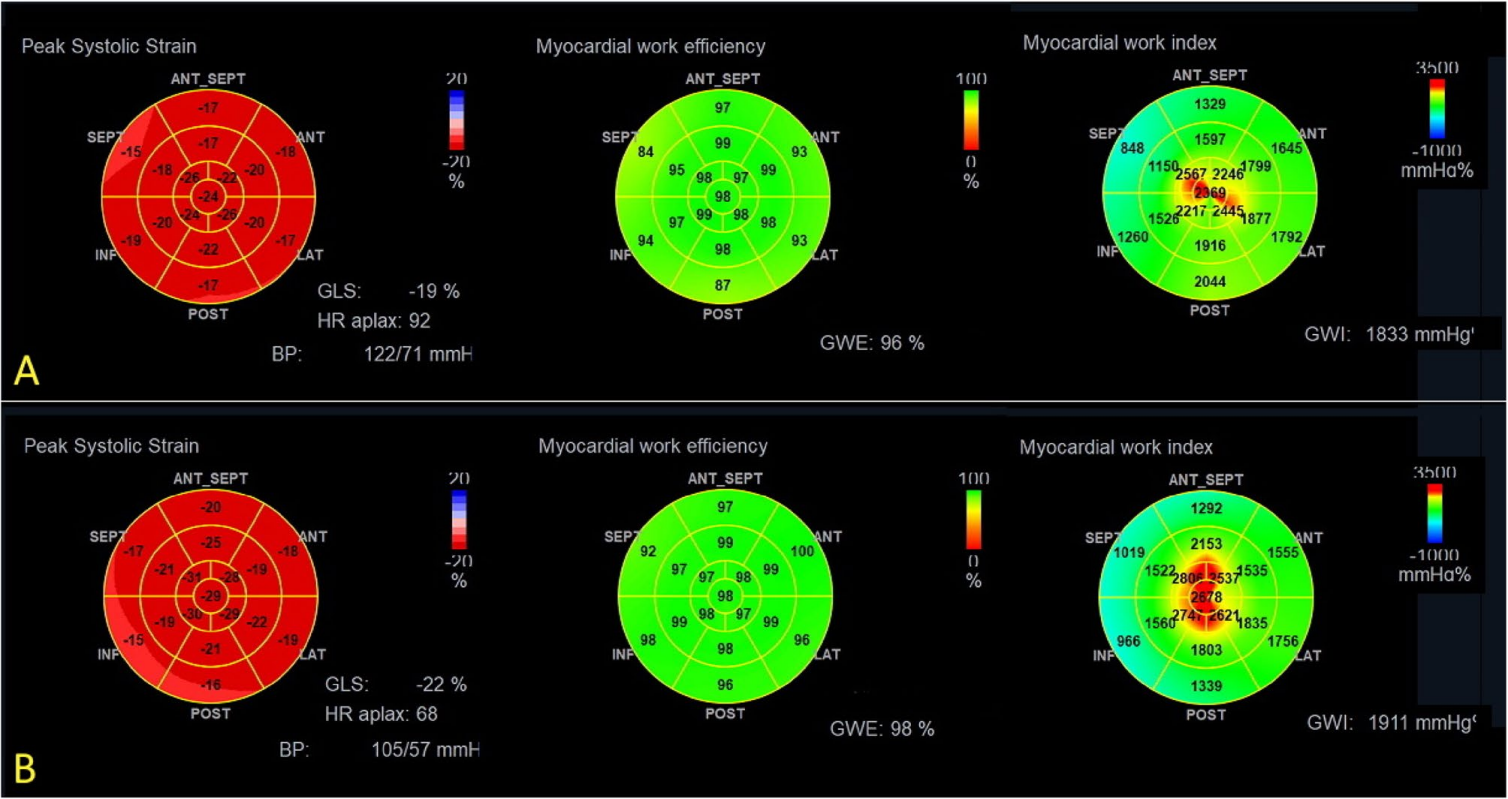
Global longitudinal strain and myocardial work index parameters before and after the HBOT in a 45-year-old patient. (**A**) Top panel. Before the treatment. From left-to-right: global longitudinal strain = − 19%, global work efficacy = 96%, global work index = 1833 mmHg%. (**B**) Bottom panel. After the treatment. From left-to-right: global longitudinal strain = − 22%, global work efficacy = 98%, global work index = 1911 mmHg%.

Post-hoc analysis was performed on the subgroup of patients with reduced GLS at baseline defined as lower than − 20% (Table 4). There were 29 (48.3%) patients with reduced GLS at baseline, with 13 (43.3%) and 16 (53.3%) patients allocated to the sham and HBOT groups, respectively, (*p* = 0.44). The mean GLS in both groups was similar at baseline. Compared to the sham group, GLS significantly increased following HBOT (− 17.8 ± 1.1 to − 20.2 ± 1.0, *p* = 0.0001). The net effect size was 0.245 with a significant group by time interaction (*p* = 0.041) (Table 4, Fig. 2). Following HBOT, 62.5% (10/16) had normalized GLS to a level of − 20 or higher, compared to 38.4% (5/13) in the sham group (*p* = 0.08). There was a significant increase in the global work efficacy following HBOT (96.3 ± 0.9 to 97.1 ± 1.1, *p* = 0.02) compared to the sham group (96.2 ± 2.1 to 96.7 ± 1.03, *p* = 0.24). However, the net effect size was 0.05 with a non-significant mixed model (*p* = 0.52) (Table 4). There were no other significant changes between the two subgroups (Table 4). Figure 3 is an example of myocardial work index parameter changes of a 45-year-old patient with reduced GLS at baseline who underwent HBOT.

**Table 4:** Myocardial work index parameter changes in patients with reduced GLS.

	HBOT (N = 16)				Sham (N = 13)				ANOVA group by time interaction		
	baseline	post	*p* value	change	Baseline	post	*p* value	change	Net size effect	F	*p* value
GLS, %	− 17.8 ± 1.1	− 20.2 ± 1.0	**0.0001**	− 2.4 ± 2.26	− 17.8 ± 1.2	− 19.1 ± 2.1	0.08	− 1.3 ± 1.8	0.245	5.234	**0.041**
GWI, mmHg'	1848.8 ± 296.4	2022.6 ± 338.8	0.16	43.8 ± 265.4	1719.5 ± 265.4	1781.7 ± 286.8	0.57	62.2 ± 219.6	0.585	1.681	0.219
GCW, mmHg'	2084.1 ± 305.7	2314.6 ± 310.1	0.06	230.5 ± 329.3	2029.9 ± 295.0	2045.2 ± 320.9	0.45	16.4 ± 225.5	0.295	4.585	0.530
GWW, mmHg'	64.1 ± 19.2	53.1 ± 22.7	0.17	− 11.1 ± 20.9	66.7 ± 46.8	55.0 ± 20.0	0.2	− 11.7 ± 43.2	0.045	4.585	.0.053
GWE, %	96.3 ± 0.9	97.1 ± 1.1	**0.02**	0.79 ± 1.1	96.2 ± 2.1	96.7 ± 1.03	0.24	0.46 ± 1.7	0.051	0.425	0.527

GLS, global longitudinal strain; GWI, global work index; GCW, global constructive work; GWW, global wasted work; GWE, global work efficacy.

Significant values are in bold.

The data were analyzed by ML blindly. Interobserver and intraobserver variabilities (ML and VT) were done on 20 random study patients and were both found to range up to 5%.

## Discussion

The current randomized controlled trial demonstrates a subtle systolic dysfunction of GLS lower than − 20%, in about half (48.3%) of the post-COVID-19 syndrome patients, which was significantly improved by HBOT. This recovery in GLS by HBOT exceeded the natural recovery rate observed in the sham group.

Previous studies have documented cardiac abnormalities in post-acute COVID-19 syndrome with transthoracic echocardiography (TTE). In 80% of patients who underwent echocardiography examination during hospitalization with COVID-19, GLS was reduced^25,26^ and changes in GLS correlated with clinical manifestations^26^. Reductions in GLS were found at the end of hospitalization^26^ and at least 4–6 weeks after the recovery from the acute illness in patients with normal ejection fractions^27^. Campuzano et al. found a significant decrease in the left ventricle systolic function, confirmed by lower GLS and LVEF in 33% of 102 patients, 4–8 weeks after the acute infection^28^. Cha et al. found a reduced LVEF in six subjects (9.37%) and 18 patients (28.12%) with diastolic dysfunction out of 325 post-acute COVID-19 patients, within the first three months after infection^29^. Other echocardiography-based studies did not find abnormalities in conventional echocardiographic clinical parameters, excluding myocardial work indices, in the first two months post-infection^30–32^. In a recent systematic review, reduced GLS was reported in 30% of post-COVID-19 patients 3–6 months from infection, evaluated using cardiac MRI^33^. Our study included a relatively young previously healthy population, in which all conventional echocardiography parameters were normal except GLS. GLS is an objective and reproducible technique for evaluating myocardial deformation and is considered a more sensitive and earlier predictor of LV systolic dysfunction compared to LVEF^34^.

GLS has also been shown to allow the detection of subclinical myocardial dysfunction due to its ability to predict early-stage myocardial fibrosis in correlation with CMR^35,36^. Thus, the use of advanced cardiac imaging may be required for accurately evaluating post-COVID-19 patients' myocardial function.

The principal pathophysiological mechanisms considered responsible for the myocardial damage caused by COVID-19 infection are related mostly to direct virus-induced injury due to a high distribution of ACE2 receptors on cardiac myocytes which are binding sites for virus particles^37,38^. Other relevant factors are a systemic inflammatory response due to the cytokine storm, hypoxia-induced oxygen supply‐demand mismatch, micro‐or macrovascular thrombosis following inflammation and endothelial dysfunction, as well as persistent cardiac inflammation in the form of peri‐myocarditis^36,38,39^. The cardiac involvement during the acute phase of COVID-19 can have a clinical impact on the long-term prognosis, including worsening of previous cardiac disease or developing new cardiac conditions. Several studies show a correlation between inflammatory markers and reduced GLS in the post-recovery from COVID‐19^27,40^.

In the past decade, novel HBOT protocols have been shown to induce both regenerative and anti-inflammatory effects^11,13–17^. These protocols, including the one used in the current study, utilize the so called “hyperoxic-hypoxic paradox”, in which repeated fluctuations in both pressure and oxygen concentrations induce gene expression and metabolic pathways that are essential for regeneration without the hazardous hypoxia^10^. These pathways can modulate the immune system, decrease systemic inflammation, promote angiogenesis, and restore mitochondrial function^10–18^. Some or all of these effects may explain the beneficial effects found in the current study. These effects on myocardial function have been previously demonstrated by our group in a clinical trial performed on healthy adults, in which HBOT induced significant increases in GLS, LVEF, and the myocardial performance index [MPi]. The main effects were seen in regional strain in the apical and anteroseptal segments^ 41^.

In the current study, GLS significantly improved following HBOT, compared to the sham protocol. However, the significant mixed model interaction was significant in the post-hoc analysis for patients with reduced GLS. In other words, the regenerative effect of HBOT was beneficial in dysfunctional cardiac tissue, compared to supposedly healthy functional cardiac tissue. This finding has been validated in studies of non-healing wounds using HBOT, as well as recently in neurological diseases with dysfunctional (but non-necrotic) brain tissue. Recently, Robbins et al. reported a significant improvement in fatigue following HBOT sessions in post-COVID-19 patients^19^. However, the effect on cardiac function was not evaluated. Our group has recently shown that HBOT can improve both cognitive function and physical and psychiatric symptoms in post-COVID-19 syndrome patients^20^. To the best of our knowledge, this is the first study that investigated the effect of hyperbaric oxygen therapy on myocardial function in post-COVID-19 patients.

The study has several strengths and limitations. The primary strength of this study is the sham protocol for the control arm. The main limitation lies in the relatively small sample size. A second limitation is that echocardiographic evaluation is operator-dependent. However, all the echocardiography examinations were performed by experienced sonographers and evaluated by the same senior cardiologists, who were blinded to the patient's allocation. Third, although the HBOT protocol included 40 sessions, an optimal number of sessions for maximal therapeutic effect has yet to be determined. Fourth, the SARS-CoV-2 different variants are not routinely tested in clinical practice, and therefore the data were unavailable as a possible co-factor. Lastly, results were collected 1–3 weeks after the last HBOT session. Long-term results as well as possible long-term clinical cardiac complications remain to be collected.

In conclusion, in post-COVID-19 patients, slightly reduced GLS despite normal EF is a frequent finding and can indicate subclinical left ventricular dysfunction. HBOT promotes cardiac systolic function recovery in patients suffering from the post-COVID-19 condition. Further studies are needed to optimize patient selection and to evaluate long-term outcomes.

## Data Availability

The datasets analyzed during the current study available from the corresponding author on reasonable request.

## Abbreviations

GLS: Global longitudinal strain
GWI: Global work index
GCW: Global constructive work
GWW: Global wasted work
GWE: Global work efficacy
